# The Question of Priority in the Introduction of the Process of Bleaching Discolored Teeth

**Published:** 1899-07

**Authors:** 


### Foreign Correspondence.

### THE QUESTION OF PRIORITY IN THE INTRODUCTION OF THE PROCESS OF BLEACHING DISCOLORED TEETH.

Berlin, Germany, Victoria Strasse, May 9, 1899.

To the Editor:

Sir,—In the January number of the International Dental Journal the editor, in reviewing my "Lehrbuch der Zahnheilkunde," took me to task for not having given him credit as the originator of the process of bleaching teeth by chlorinated lime and acetic acid, stating at the same time that it is the habit of nearly all dental writers to ignore the important work which he has done. I wish to assure Dr. Truman that this omission was no more intentional on my part than probably on the part of the other writers to whom he refers. We of the dental profession are all too much indebted to Dr. Truman for his life-long efforts towards the advancement of dental science to willingly do anything to detract from his well-earned reputation. In the case of bleaching teeth with chlorinated lime, it is about the same as in all other discoveries or inventions which have become common property.

We go on enjoying the benefits resulting from such discoveries without thinking of the discoverer or of his too often unrequited labors. I shall make good the omission in the next edition of my book (should there be one) to Dr. Truman, as well as to the originators of the other methods of bleaching.

In this connection I wish to call attention to an error which crept into the review and which I would like to correct. I am reported as saying, "Of all the remedies which have become practical in pulpitis, I regard iodoform as the best." This is not quite accurate. I really said, "Of the remedies which are often used in pulpitis, I *mention* iodoform first." I have not treated a case of pulpitis with iodoform for ten years.

W. D. MILLER.

REMARKS.

While appreciating the kindly intentions of Professor Miller, the editor of this journal would not be misunderstood in regard to the criticism alluded to. It may seem to the general reader that Dr. Truman is peculiarly sensitive in regard to the paternity of the processes originating with him. This would be very far from the truth. Whatever work he has done, whether that be much or little, has been to make the dental profession better in its practical work, and not to build any monument for himself. He, however, regards the facts of dental history important. The want of care in the compiling of books has made the work of the dental historian exceedingly difficult. It is a great labor to search for the originator of any method of practice, but if the author fails to do this, he simply increases the difficulty for those who succeed him, if he does not efface the originator entirely from the minds of the profession.

The matter would not have been alluded to in the review, but for the fact that several books have been issued in recent years all equally guilty in this respect. It is true that the dental profession "go on enjoying the benefits resulting from such discoveries," and not only do not thank the discoverer, whoever he may be, but quietly weave into papers the results attained, and thus deprive the originators of the only remuneration for their work expected or desired, —credit for that accomplished.

It is with regret that the review over-stated Professor Miller's views in regard to iodoform. The impression conveyed to the reviewer's mind was that Professor Miller regarded this agent as the best to use in pulpitis. He will accept, with this explanation, also the regret that he has not used this drug in "ten years," for he has thereby lost, in the opinion of the writer, the aid of one of the best, if not *the* best, agent in use, not so much as an antiseptic but as an analgesic.—Ed.

